# Utilization of steric hindrance of alkyl lithium-based initiator to synthesize high 1,4 unit-containing hydroxyl- terminated polybutadiene

**DOI:** 10.1098/rsos.180156

**Published:** 2018-05-30

**Authors:** Xin Min, Xiaodong Fan, Jie Liu

**Affiliations:** Northwestern Polytechnical University, Xi'An, Shannxi 710000, People's Republic of China

**Keywords:** mono-lithium-based initiator, steric hindrance, anionic polymerization, hydroxyl-terminated polybutadiene, 1,4 unit content

## Abstract

A novel alkyl lithium-based initiator with relatively large steric hindrance, *tert*-butyldimethylsiloxydimethylpropyl lithium (TBDMSODPrLi), was designed and synthesized. By using TBDMSODPrLi, hydroxyl-terminated polybutadiene (HTPB) was prepared via anionic polymerization. The macromolecular structure of HTPB was characterized and verified by FTIR and ^1^H-NMR. It was found that 1,4 unit content in HTPB initiated by TBDMSODPrLi was significantly higher (over 90%) compared to a HTPB (1,4 unit content of 70%) initiated with another initiator possessing smaller steric hindrance. The possible mechanism, which was based on initiator steric hindrance affecting monomer chain addition behaviour, was deduced. It was that the initiator's larger steric hindrance blocked lithium's intermolecular association during anionic polymerization; as a result, it could effectively increase the 1,4 unit content in HTPB. To further study how to obtain higher and stable 1,4 unit content, the optimal anionic polymerization technique for HTPB was explored including polymerization temperature, time and the amount of initiator used. The study concluded that utilization of an initiator with larger steric hindrance and reducing the polymerization temperature were two important factors to raise the 1,4 unit content in HTPB.

## 1. Introduction

Hydroxyl-terminated polybutadiene (HTPB) is an important liquid rubber with high transparency, low viscosity, ageing resistance and excellent low-temperature mechanical properties. Currently, HTPB's industrial production mainly relies on free radical polymerization, but with such a synthesis the HTPBs present many shortcomings such as higher molecular weight distribution and difficulty to control the molecular weight, especially the lower 1,4 unit content. It is well known that besides the molecular weight and its distribution, the 1,4 unit content in HTPB is a crucial structural parameter which can strongly affect the physical properties of the commercial products.

**Figure 1.**
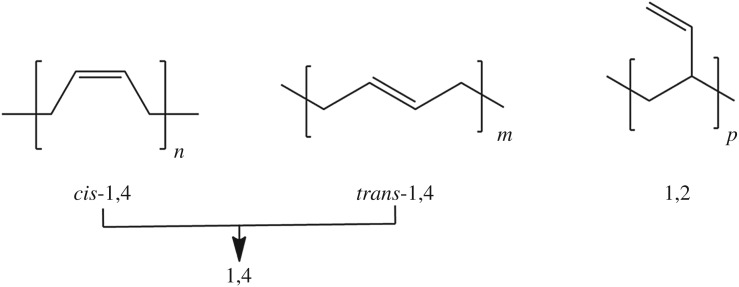
The segment microstructures in HTPB.

1,4 unit refers to the sections of polymer chain that are connected by 1-C and 4-C of butadiene monomers. The glass transition temperature of 1,4 unit is −106°C, which means that the segment still maintains flexibility at extremely low temperature. There are two kinds of configuration of 1,4 unit (as shown in figure 1) which are called *cis*-1,4 and *trans*-1,4. Whereas *trans*-1,4 unit is crystallized easily due to the aligned chain segment structure, the crystallization phenomenon of *cis*-1,4 was hard to observe in normal conditions. 1,2 unit refers to the sections of polymer chain which are connected by 1-C and 2-C of butadiene monomers. It has a higher glass transition temperature (*T*_g_= 15°C). The content of 1,2 unit in HTPB can seriously affect the mechanical properties of HTPB, especially at low temperature. To increase the 1,4 unit content of HTPB, anionic polymerization was often used. To date, the 1,4 unit content in HTPB via anionic polymerization could usually reach 86% [1]; however, this number is still not satisfactory for many related industries which need HTPBs with much higher 1,4 unit content.

**Figure 2.**
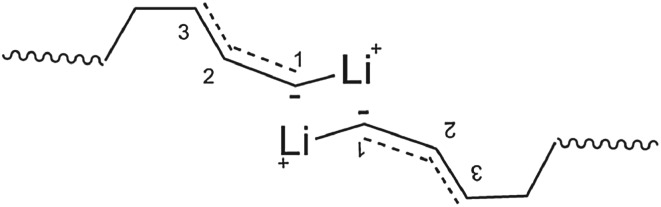
Association effect of alkyl lithium-based initiator.

Besides the use of advanced synthesis technology to produce higher 1,4 unit-containing HTPB, the development of novel initiators is another main trend of research. Among them, alkyl lithium-based initiators are a hot direction. O'Driscoll [2] reported that only the unassociated alkyl lithium initiator had catalytic activity in non-polar solvent, which might cause the efficiency problem of initiator. Sinn *et al.* [3] suggested that there was no unassociated alkyl lithium when the concentration of alkyl lithium was more than 10^−3^ mol l^−1^, which indicated that the associated alkyl lithium was the main active centre under this circumstance. Natta [4] indicated that 1,2 structure of butadiene polymerized by *tert*-butyl lithium increased from 7% to 47% when the concentration of *tert*-butyl lithium concentration increased from 0.005 mol l^−1^ to 0.5 mol l^−1^. The literatures also indicated that the associated alkyl lithium is not beneficial to the production of 1,4 unit. To summarize the current research results, it was concluded that because of the association of end chain of lithium-based initiator, 1-C atom had to be embedded in the centre of the association, and the monomer could not get close to the 1-C position. As a result, the process leads to form 1,2 unit configuration. The detailed mechanism of molecular association is shown in figure 2.

The reduction of the association is an effective method to markedly raise 1,4 unit content via functional initiator. Usually, there are two methods to block the initiator's association: one is addition of Lewis alkali such as tetrahydrofuran (THF). But, this method can weaken the binding force between carbon anion and lithium, the ability to control the end chain is reduced, and the content of 1,4 unit could not be effectively increased. The other method is to increase the steric hindrance of alkyl lithium initiator, and utilize the geometrical size of the initiator to stop end chain association.

Generally speaking, high branching coefficient of alkyl lithium leads to high steric hindrance, so the degree of association of alkyl lithium can be reduced with the increase in branching coefficient. Bates and colleagues [5,6] synthesized TIPSODMPrLi with triisopropylsiloxy as the steric hindrance group, but triisopropylsiloxy was far away from the Li atom, and the effect of steric hindrance could not be exhibited sufficiently. Furthermore, TIPSODMPrCl, the precursor of TIPSODMPrLi, was too expensive to realize industrialization. It needs at least 22 h to synthesize the initiator to guarantee the conversion of TIPSODMPrCl, which is inefficient for industrialization. Based on this analysis, in this article, a novel initiator, *tert*-butyldimethylsiloxydimethylpropyl lithium (TBDMSODPrLi), with high steric hindrance was designed and synthesized by low-cost materials in an efficient way. In cooperation with methyl in the structure of the initiator which was close to the Li atom, the branching coefficient was markedly increased leading to an increase in the steric hindrance of the initiator. As a result, the end chain association could be effectively avoided during polymerization. To verify the theory discussed above, another initiator, 4-methoxy-1-butyllithium, was synthesized to compare with TBDMSODPrLi via ^1^H-NMR data. To select a stable and systematic technique for carrying out HTPB preparation, the preparation process, including the effect of polymerization temperature, time and amount initiator used, was also explored, and finally, an optimal synthesis technique was determined.

## 2. Experimental

### 2.1. Materials

Butadiene (15 wt% in *n*-hexane), TCI; 3-chloro-2,2-dimethyl-1-propanol (99%), J&K; imidazole (99%), J&K; lithium (99.5%), Acros; tetrabutylammonium fluoride (1 mol l^−1^ in THF), TCI; toluene, AR; *n*-hexane, AR; cyclohexane, AR; benzene, AR; THF, AR; *N,N*-dimethylformamide (DMF), AR; methanol, AR; hydrochloric acid, AR.

All the solvents used in the experiment were purified according to the following procedure. Firstly, hydrogenated calcium was added to solvents. Secondly, decompression distillation and specific temperature fraction were employed. Thirdly, molecular sieves were added when the solvents were stored. Fourthly, solvents were purified with high purity argon to remove the trace air.

### 2.2. Synthesis of precursor TBDMSODPrCl

*Tert*-butyldimethylchlorosilane (15.07 g, 0.1 mol) and 3-chloro-2,2-dimethyl-1-propanol (12.26 g, 0.1 mol) were dissolved in a flask, then DMF (50 ml) was added as the solvent. Imidazole (8.84 g, 0.13 mol) was used as the catalyst. The mixture was warmed to 60°C and stirred for 3 h. During this time, the reaction was traced with thin-layer chromatography until the feedstock point disappeared. The mixture was filtered through Celite to remove the salt of imidazolium, the coarse product was extracted three times with *n*-hexane, and washed three times using water. Magnesium sulfate anhydrous was used to dry the product, and the solvent was evaporated under vacuum. A silicon-based gel column was employed to purify the product, and petroleum ether was used as an eluent. Finally, *tert*-butyldimethylsiloxydimethylpropyl chloride (TBDMSODPrCl) was obtained and the yield was 98%. ^1^H-NMR (400 MHz, CDCl_3_, *δ*): 0.07 (s, 6 H; CH_3_), 0.92 (s, 9 H; CH_3_), 0.97 (s, 6 H; CH_3_), 3.38 (s, 2 H; CH_2_), 3.46 (s, 2 H; CH_2_).

### 2.3. Synthesis of initiator TBDMSODPrLi

Standard Schlenk techniques were used to control all moisture and air-sensitive compounds under a high-purity argon atmosphere. TBDMSODPrCl (11.8 g, 0.05 mol) was dissolved in 50 ml cyclohexane under argon atmosphere. In a dry flask, 150 ml cyclohexane and 3.5 g lithium were added under argon atmosphere, and the system was warmed to 50°C. During this time, the cyclohexane solution of TBDMSODPrCl was added dropwise under argon atmosphere. The mixture was warmed to 70°C and stirred for 6 h. After the solution became turbid, the mixture was cooled down to room temperature. The mixture was concentrated under argon atmosphere and filtered to remove the salt. TBDMSODPrLi initiator product was kept in the cyclohexane solution under a sealed condition. Because the solution of alkyl lithium-based initiator was sensitive to air and water, it also needed to be preserved at low temperature under argon atmosphere.

### 2.4. Synthesis of 4-methoxy-1-butyllithium

4-Methoxy-1-butyllithium was synthesized according to the literature [7].

### 2.5. Synthesis of hydroxyl terminated polybutadiene

All experiments were performed with Schlenk technique. Butadiene solution (10 ml, 15 wt% in *n*-hexane) was added in a Schlenk bottle. TBDMSODPrLi (0.5 mol l^−1^ in cyclohexane) was added under argon atmosphere and stirred for several hours at room temperature, during which time the colourless solution turned yellow. After several hours, 0.2 ml epoxy ethane was added and stirred for 1 h, and then 0.5 ml of methanol was added to terminate reaction. The solvent was concentrated via vacuum. The polymer was again dissolved in THF, and washed 3 times with 300 ml methanol. The polymer product as a colourless and transparent viscous liquid was obtained. The yield was 98%.

Dry HTPB (1 g) was dissolved in 10 ml THF, and then hydrochloric acid (1 ml) was added. The mixture was stirred at 50°C for 4 h. After that, the solvent was removed under vacuum (vacuum degree: 0.08 MPa) and HTPB was re-dissolved in THF, and washed three times with 300 ml methanol. Finally, a colourless and transparent viscous liquid was obtained. The yield was 99%.

### 2.6. Measurements

(1) A Nicolet iS10 Fourier transform infrared (FTIR) spectrometer (Thermo Fisher Scientific) was used to obtain the infrared spectra for both low molecular weight compounds and polymers. Sample preparations were conducted as follows: 2 ml CH_2_Cl_2_ solution of HTPB or low molecular weight compounds (2 mg ml^−1^) were coated on KBr tablets and dried at room temperature and pressure; this way can avoid thick coatings to obtain correct data. Scanning range: 500–4000 cm^−1^, resolution: 4 cm^−1^, scanning time: 16.
(2) Bruker Avance-400 NMR was used to obtain the ^1^H-NMR and ^13^C-NMR spectra for both precursor and polymer at room temperature. CDCl_3_ was used as the solvent; internal standard was selected as tetramethylsilane.
(3) SEC-MALL was used to measure the molecular weight and molecular weight distribution of polymers at room temperature. Mobile phase: chromatographic grade THF, flow rate: 0.5 ml min^−1^, concentration of sample solution: 10 mg ml^−1^, sample amount: 200 µl.

#### 2.6.1. Calculation method

The contents of 1,4 unit and 1,2 unit in HTPB were calculated through ^1^H-NMR data by using the following equation. The results were also verified via FTIR data using equation (2.2).

2.1 $$B\%=\left(1-\frac{2I_{\left(5.01∼4.8\right)}}{2I_{\left(5.38\right)}+I_{\left(5.01∼4.8\right)}}\right)\times 100\text{\%,}$$

where *I* is the integral area of ^1^H-NMR of HTPB at certain chemical shift and *B* is the content of 1,4 structure in HTPB.

2.2 $$\begin{array}{rl} C_{c}\% & \;=\frac{D_{724\;cm^{-1}}/K}{D_{724\;cm^{-1}}/K_{c}+D_{911\;cm^{-1}}/K_{v}+D_{967\;cm^{-1}}/K_{t}}\times 100\%,\\ C_{v}\% & \;=\frac{D_{911\;cm^{-1}}/K_{v}}{D_{724\;cm^{-1}}/K_{c}+D_{911\;cm^{-1}}/K_{v}+D_{967\;cm^{-1}}/K_{t}}\times 100\%\\ \text{and}\;C_{t}\% & \;=\frac{D_{967\;{\mathrm{cm}}^{-1}}/K_{t}}{D_{724\;{\mathrm{cm}}^{-1}}/K_{c}+D_{911\;{\mathrm{cm}}^{-1}}/K_{v}+D_{967\;{\mathrm{cm}}^{-1}}/K_{t}}\times 100\%, \end{array}\}$$

where *D* is the absorbance of the corresponding band in the FTIR spectrum, *K_c_* the absorption coefficient of *cis*-1,4 unit at 720–740 cm^−1^, *K_v_* the absorption coefficient of 1,2 unit at 911 cm^−1^, *K_t_* the absorption coefficient of the *trans*-1,4 unit at 967 cm^−1^, and *C_c_%*, *C_v_%* and *C_t_%* are the contents of *cis*-1,4 unit, 1,2 unit and *trans*-1,4 unit, respectively.

The concentration of lithium element in TBDMSODPrLi was measured by following standards of the People's Republic of China (non-ferrous metals industry standard YS/T 830-2012).

## 3. Results and discussion

### 3.1. Synthesis of precursors TBDMSODPrCl and TBDMSODPrLi

**Figure 3.**
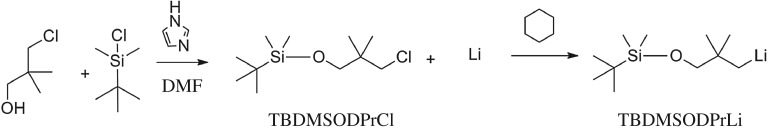
Synthesis routes of alkyl lithium initiator TBDMSODPrLi.

The synthesis routes of both TBDMSODPrCl and TBDMSODPrLi are shown in figure 3. To increase the branching coefficient of initiator, methyl was introduced in the structure of the initiator, which could raise the steric hindrance of initiator and reduce the end chain association. Precursor TBDMSODPrCl was characterized by ^1^H-NMR as shown in the supporting information. The chemical shifts at 0.92 and 0.97 ppm were the characteristic peaks of both *tert*-butyl and methyl in halohydrocarbon. Therefore, it indicated that the precursor TBDMSODPrCl had been successfully synthesized.

**Figure 4.**
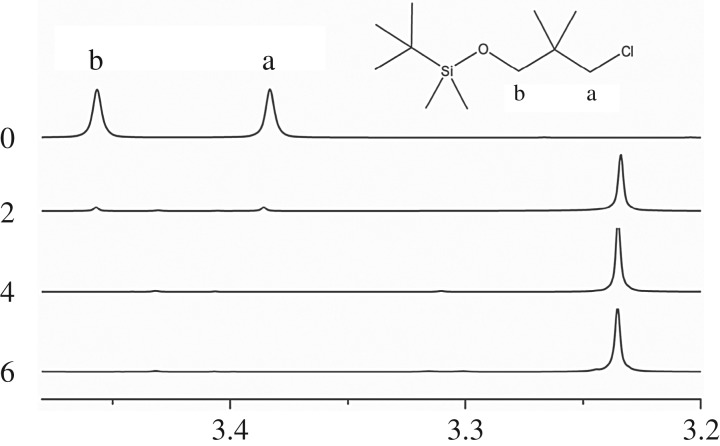
Tracking ^1^H-NMR spectrum of TBDMSODPrLi after termination by methyl alcohol.

The reaction between halohydrocarbon and lithium was adopted as a routine way. To completely replace CI atoms, not only should the amount of metal lithium be in excess, but also the grain metal lithium should be small enough under the argon atmosphere. Because alkyl lithium is unstable in air, it must be kept in solution, and, therefore, caused difficulty to directly characterize via a scientific instrument, so the reaction was traced by ^1^H-NMR after termination by methyl alcohol (shown in figure 4). In this case, the concentration of alkyl lithium was characterized by chemical titration with 2,2'-dipyridyl as the indicator.

### 3.2. Polymerization mechanism of polybutadiene initiated by TBDMSODPrLi

**Figure 5.**
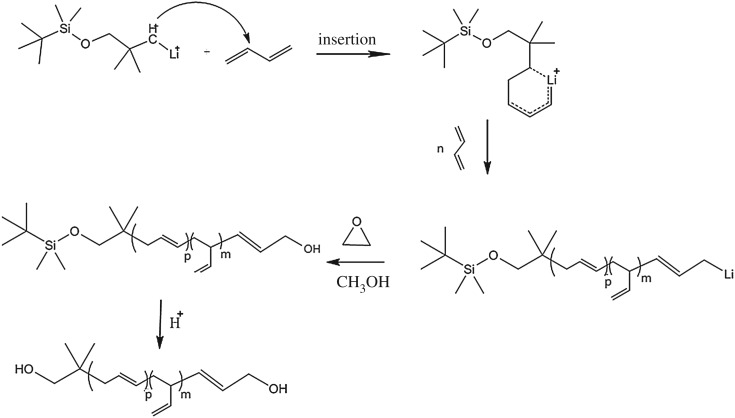
The initiation mechanism of alkyl lithium initiator TBDMSODPrLi.

The polymerization mechanism of butadiene initiated via TBDMSODPrLi was a typical anionic style. The possible process of chain addition and propagation is shown in figure 5. Based on the fundamental theories [4], the carbon anion would first attack butadiene, and a temporal intermediate state of six-member ring could be formed, with which active centres were established for continuously triggering activity of butadiene monomer. The way of 3-C position being attacked by butadiene could be blocked via steric hindrance of initiator; as a result, 1,2 insertion forms of chain propagation were reduced. At the same time, 1,4 unit insertions were enhanced by alkyl lithium initiator attacking 1-C position of butadiene. After monomers were converted completely, the epoxy ethane was added to initiate ‘capped’ reaction, and lithium alkoxy structures were formed. Double hydroxyl-terminated polybutadiene could finally be obtained by hydrolysis under an acid condition.

During polymerization, it was found that the addition of polar additives, such as THF, could cause a serious reduction of 1,4 unit content in HTPB. This is because most polar materials are Lewis alkalis, which contain a couple of lone pair electrons, and lone pair electrons could coordinate with lithium, and make the lithium atom form a free ion; as a result, polar materials weakened lithium's controlling effect on end chains, and reduced 1,4 unit content. This conclusion was consistent with Chen's [1].

### 3.3. Calculation of 1,4 unit content in hydroxyl terminated polybutadiene

**Figure 6.**
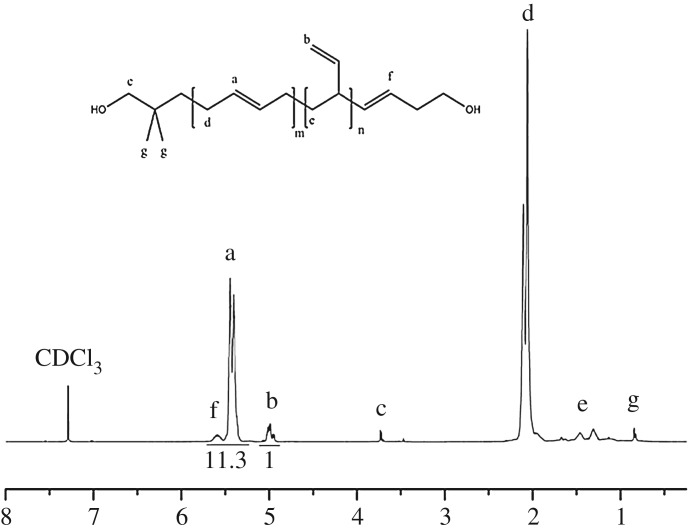
^1^H-NMR spectrum of HTPB containing high 1,4 content.

The 1,4 unit content in HTPB was measured and calculated via ^1^H-NMR and FTIR techniques. As shown in figure 6, ^1^H-NMR spectrum of HTPB initiated by TBDMSODPrLi was collected, and the calculation was done according to equation (2.1). In this case, the chemical shift around 5.5 which represented as 1,4 unit was integrated as 11.3, and the chemical shift around 5 which represented as 1,2 unit was also integrated as 1. By using equation (2.1), the final result of the calculation of 1,4 unit content in HTPB was 91.5%.

**Figure 7.**
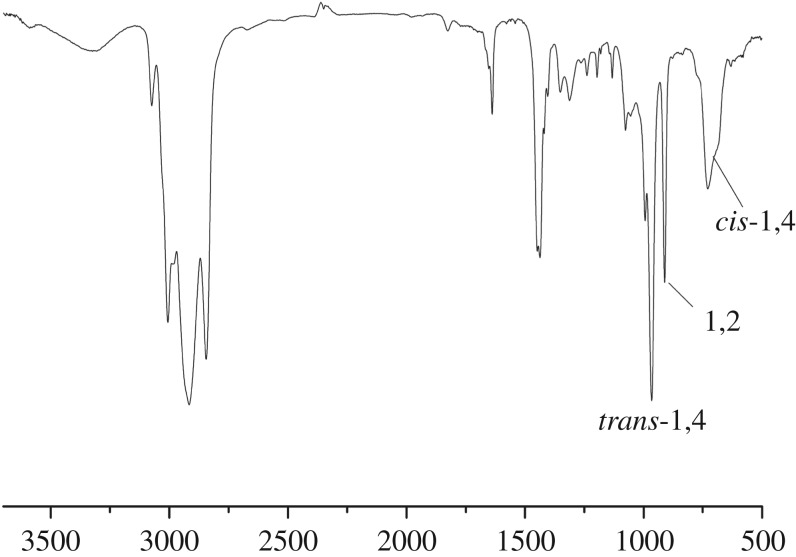
FTIR spectrum of HTPB containing high 1,4 content.

The FTIR spectrum was used to further confirm the calculation result of ^1^H-NMR. The resulting data are shown in figure 7. Evidently, through the calculation via FTIR, *cis*-1,4unit was 35.2%, *trans*-1,4 unit was 56.3% and 1,2 unit was 8.5%. Here, it must be noted that this result might imply that the effect of steric hindrance of initiator was not significant to affect the content of *cis*-1,4 unit in HTPB. The main reason may rely on Li metal atoms of the initiator; the electronic absorption of Li was not strong enough to effectively control the configuration of end chain to form *cis*-1,4 unit.

### 3.4. Comparison of structural influence on 1,4 unit content between TBDMSODPrLi and 4-methoxy-1-butyllithium

**Figure 8.**
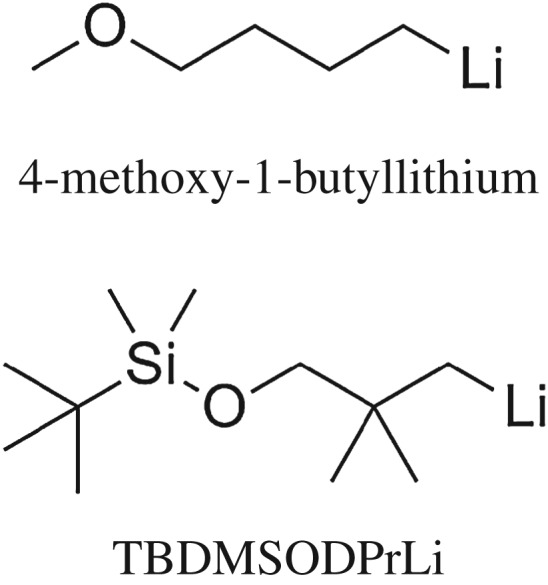
Molecular structures of 4-methoxy-1-butyllithium and TBDMSODPrLi.

**Figure 9.**
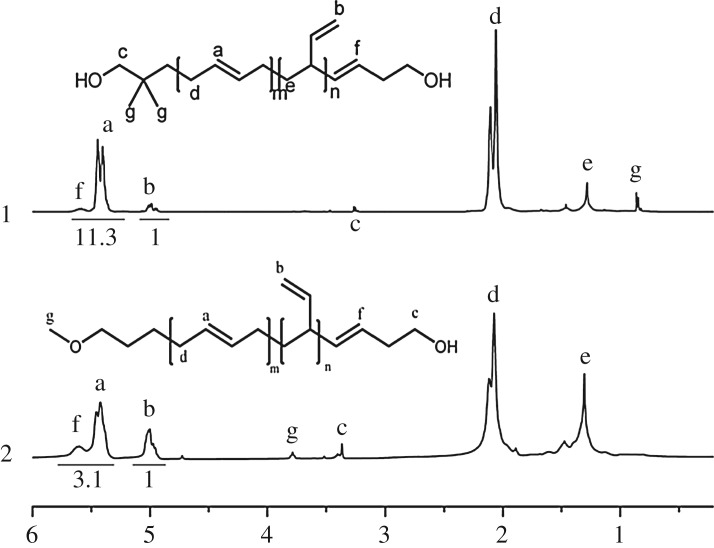
Comparison of ^1^H-NMR spectral data between TBDMSODPrLi and 4-methoxy-1-butyllithium. Note: 1 refers to the ^1^H-NMR of HTPB initiated by TBDMSODPrLi; 2 refers to the ^1^H-NMR of HTPB initiated by 4-methoxy-1-butyllithium.

To verify theoretical analysis above, that is, the steric hindrance of the initiator could increase the 1,4 unit content in HTPB, two different initiators, TBDMSODPrLi and 4-methoxy-1-butyllithium [7], were synthesized and used to initiate the anionic polymerization of butadiene monomer. Their structural differences are presented in figure 8. At the same time, the detailed 1,4 unit structural characterization data are shown in figure 9. Evidently, only from a comparison of geometrical size between the two compounds, it was found that the steric hindrance of TBDMSOPrLi was higher than that of 4-methoxy-1-butyllithium.

After calculation using equation (2.1), the content of 1,4 unit in HTPB initiated by 4-methoxy-1-butyllithium was 72.4% and the ^1^H-NMR data for TBDMSODPrLi was 91.5% as shown in table 1. Interestingly, even 4-methoxy-1-butyllithium could not raise the 1,4 unit content; its synthesized molecular weight and molecular distribution still tightly followed an anionic polymerization mechanism. Here once again, it was proved that the initiator's steric hindrance could indeed raise the 1,4 unit content in HTPB. The result also indicated that the designed and synthesized novel initiator, TBDMSODPrLi, was a reliable and effective initiator which could be potentially used as a good initiator to the HTPB industry.

**Table 1.** The data of 1,4 unit content, *M*_n_ and *M_w_/M_n_* of HTPB synthesized via two initiators. Note: amount of catalysts was 2.5 × 10^−4^ mol; butadiene (15 wt% in *n*-hexane): 10 ml; solvent: *n*-hexane; polymerization time: 4 h, polymerization temperature: 50°C.

initiator	1,4 content (%)	*M*_n_ (g mol^−1^)	*M*_w_/*M*_n_
4-methoxy-1-butyllithium [7]	72.4	4080	1.10
TBDMSODPrLi	91.5	4046	1.11

### 3.5. Exploration of optimal polymerization process of hydroxyl terminated polybutadiene

To explore an efficient polymerization process in which the 1,4 unit content including molecular weight and its distribution was totally targeted as the main parameters, TBDMSODPrLi was used as the initiator and butadiene was used as the monomer to conduct the process experiment.

Table 2 shows that monomer conversion was increased gradually upon raising polymerization temperature. But, this trend of conversion became not significant after the polymerization temperature reached to over 50°C, and at this temperature range, the molecular weight distribution became wide accordingly. The chain transfer and chain termination caused by such a high polymerization temperature might be the main reasons for enlarging molecular weight distribution.

**Table 2.** Effect of polymerization temperature on structural parameters of HTPB.

temperature (°C)	amount of TBDMSODLi (0.5 mol l^−1^ in cyclohexane) (mol)	amount of butadiene (15 wt% in *n*-hexane) (ml)	solvent	polymerization time (h)	*M*_n_ (g mol^−1^)	*M*_w_/*M*_n_	1,4 content (%)
30	2.5 × 10^−4^	10	*n*-hexane	4	3726	1.11	92.0
40	2.5 × 10^−4^	10	*n*-hexane	4	4026	1.12	91.9
50	2.5 × 10^−4^	10	*n*-hexane	4	4050	1.09	91.5
60	2.5 × 10^−4^	10	*n*-hexane	4	4046	1.20	90.8

Table 2 also suggests a fact that low polymerization temperature might be beneficial to increase in the content of 1,4 unit. Because the low temperature could reduce the movement of the end chains, which could increase the controlling force of Li element on the end chain, the polymer's 1,4 unit content was augmented. It could be concluded that 50°C is a more economic and efficient polymerization temperature for synthesizing HTPB.

Table 3 shows the effect of polymerization time on structural parameters of HTPB. Clearly, at the beginning, the molecular weight of HTPB gradually increased, but when the time was extended over 4 h, the molecular weight was not subject to much increase. The 1,4 unit content and molecular weight distribution were also not changed significantly. The result accorded well to the living polymerization mechanism with which fast trigger, slow growth and the degree of polymerization were increasing gradually with the time. Therefore, the effect of polymerization time on the 1,4 unit content and molecular weight as well as molecular weight distribution was not great. Finally, the optimal polymerization time should be 4 h.

**Table 3.** Effect of polymerization time on structural parameters of HTPB.

time (h)	amount of TBDMSODLi (0.5 mol l^−1^ in cyclohexane) (mol)	amount of butadiene (15 wt% in *n*-hexane) (ml)	solvent	polymerization temperature (°C)	*M*_n_ (g mol^−1^)	*M*_w_/*M*_n_	1,4 content (%)
2	2.5 × 10^−4^	10	*n*-hexane	50	3428	1.11	91.4
3	2.5 × 10^−4^	10	*n*-hexane	50	4024	1.11	91.4
4	2.5 × 10^−4^	10	*n*-hexane	50	4035	1.09	91.6
5	2.5 × 10^−4^	10	*n*-hexane	50	4046	1.12	91.6

Table 4 shows the effect of amounts of TBDMSODPrLi used in the synthesis of HTPB. It was found that the molecular weight of HTPB was increased proportionally with a reduction in amounts of initiator used. This trend was consistent with a living polymerization well indicating that HTPB's molecular weight could be controlled accurately by controlling the ratio between initiator and monomer. Reducing the amounts of initiator could slightly increase the 1,4 content because the increase in numbers of the initiators could also increase the proportion of molecular association among initiators.

**Table 4.** The effect of initiator amount on the performance parameter of HTPB.

amount (mol)	amount of butadiene (15 wt% in *n*-hexane) (ml)	solvent	polymerization temperature (°C)	polymerization time (h)	*M*_n_ (g mol^−1^)	*M*_w_/*M*_n_	1,4 content (%)
1.25 × 10^−4^	10	*n*-hexane	50	4	8043	1.11	91.8
2.5 × 10^−4^	10	*n*-hexane	50	4	4046	1.11	91.6
5 × 10^−4^	10	*n*-hexane	50	4	2026	1.11	90.2
10^−3^	10	*n*-hexane	50	4	1035	1.09	86.3

## 4. Conclusion

A novel alkyl lithium-based initiator, TBDMSODPrLi, was designed and synthesized for investigating how the steric hindrance of an initiator could influence the 1,4 unit content in HTPB during anionic polymerization. To increase the initiator's steric hindrance, the introduction of two methyl groups to the initiator was an efficient technique. Utilizing TBDMSODPrLi as an initiator, the possible mechanism of the butadiene chain initiation and propagation was studied. By comparing two initiators with different steric hindrance to initiate polymerization of butadiene, it was found that the initiator with large steric hindrance could effectively block the end chain association and increase the 1,4 unit content in HTPB. An optimal anionic polymerization process was explored and determined for HTPB, where the polymerization temperature and time were 50°C and 4 h, respectively. To accurately control the molecular weight and the 1,4 unit content of HTPB polymer, the amount of initiator should also be carefully controlled.

## Data accessibility

This article does not contain any additional data.
